# Comprehensive analysis of m^6^A methylome alterations after azacytidine plus venetoclax treatment for acute myeloid leukemia by nanopore sequencing

**DOI:** 10.1016/j.csbj.2024.02.029

**Published:** 2024-03-02

**Authors:** Zaifeng Zhang, Lili Zhang, Jiangtao Li, Ru Feng, Chang Li, Ye Liu, Gaoyuan Sun, Fei Xiao, Chunli Zhang

**Affiliations:** a The Key Laboratory of Geriatrics, Beijing Institute of Geriatrics, Institute of Geriatric Medicine, Chinese Academy of Medical Sciences, Beijing Hospital/ National Center of Gerontology of National Health Commission, Beijing, China; b Graduate School of Peking Union Medical College, Chinese Academy of Medical Sciences, 9 DongDan Santiao, Beijing 100730, China; c Clinical Biobank, Beijing Hospital, National Center of Gerontology, National Health Commission, Institute of Geriatric Medicine, Chinese Academy of Medical Sciences, Beijing, China; d Department of Hematology, Beijing Hospital, National Center of Gerontology, National Health Commission, Institute of Geriatric Medicine, Chinese Academy of Medical Sciences, Beijing, China

**Keywords:** Acute myeloid leukemia, N^6^-methyladenosine, Nanopore direct RNA-seq, Prognosis

## Abstract

N6 adenosine methylation (m^6^A), one of the most prevalent internal modifications on mammalian RNAs, regulates RNA transcription, stabilization, and splicing. Growing evidence has focused on the functional role of m^6^A regulators on acute myeloid leukemia (AML). However, the global m^6^A levels after azacytidine (AZA) plus venetoclax (VEN) treatment in AML patients remain unclear. In our present study, bone marrow (BM) sample pairs (including pre-treatment [AML] and post-treatment [complete remission (CR)] samples) were harvested from three AML patients who had achieved CR after AZA plus VEN treatment for Nanopore direct RNA sequencing. Notably, the amount of m^6^A sites and the m^6^A levels in CR BMs was significantly lower than those in the AML BMs. Such a significant reduction in the m^6^A levels was also detected in AZA-treated HL-60 cells. Thirteen genes with decreased m^6^A and expression levels were identified, among which three genes (*HPRT1*, *SNRPC*, and *ANP32B*) were closely related to the prognosis of AML. Finally, we speculated the mechanism via which m^6^A modifications affected the mRNA stability of these three genes. In conclusion, we illustrated for the first time the global landscape of m^6^A levels in AZA plus VEN treated AML (CR) patients and revealed that AZA had a significant demethylation effect at the RNA level in AML patients. In addition, we identified new biomarkers for AZA plus VEN-treated AML via Nanopore sequencing technology in RNA epigenetics.

## Graphical Abstract

**Figure:**
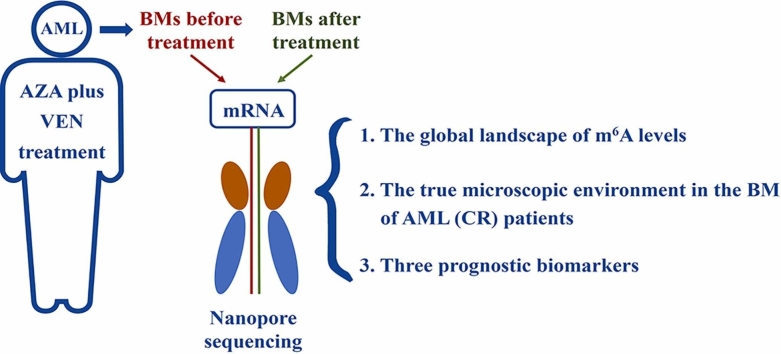

## 1 Introduction

Acute myeloid leukemia (AML) is a complex hematological malignancy characterized by hyperproliferation of hematopoietic stem cells. It has a high mortality rate, especially in elderly patients [1], [2]. According to US National Comprehensive Cancer Network (NCCN), more than half of AML patients are ≥ 65 years old, and the prevalence of AML is rising as society ages [3]. Traditionally, AML is mostly treated with intensive chemotherapy, which is often accompanied by a series of toxicities and poor outcomes and is not suitable for elderly patients [4], [5]. Therefore, novel therapeutics for AML are urgently needed.

VEN plus AZA (VEN/AZA) is an emerging therapy that can regulate epigenetics, which refers to stably heritable phenotypes resulting from changes in a chromosome without alterations in the DNA/RNA sequence (e.g., DNA 5-methylcytosine and RNA N6-methyladenosine) [6], and has been widely used in the clinical treatment of AML. AZA plays a predominant role in demethylation, acting on DNA, RNA, and protein metabolism [7]. However, the effect of AZA treatment on RNA methylation levels is undetermined in AML.

N6 adenosine methylation (m^6^A), by which 0.1%− 0.4% of all adenosine nucleotides in mammals are modified, is one of the most abundant internal markers of mammalian mRNA and has been identified as a reversible RNA methylation that alters the post-transcriptional control of gene expression and protein production [8]. The m^6^A methyltransferase complex (writers), m^6^A demethylases (erasers), and m^6^A-binding proteins (readers) dynamically manage the biological processes affected by m^6^A modifications [9]. It has been reported that there is a correlation between m^6^A modification and AML progression [10]. METTL3 (methyltransferase-like 3, writer), the most important m^6^A methylase, is abnormally activated in leukemia as studies have shown that overexpression of METTL3 can lead to enhanced proliferation and self-renewal abilities of leukemia stem cells and increase the m^6^A level in the promoter and enhancer regions, resulting in tumor development and progression [11], [12]. In patients with leukemia, the overexpression of fat mass and obesity-associated protein (FTO, eraser), a demethylase of m^6^A, may lead to abnormal increase of m^6^A level and promote the proliferation of cancer cells [13]. Furthermore, depletion of YTH N6-Methyladenosine RNA Binding Protein F1 (YTHDF1), an m^6^A reader protein, can attenuate the self-renewal, proliferation, and leukemic capacity of primary human AML cells [14]. However, since m^6^A modification is a dynamic process governed by a variety of regulators, it is difficult to clarify the true role of m^6^A in a specific disease from a single regulator. Therefore, quantifying the global landscaped of m^6^A modifications in AML after AZA/VEN treatment is crucial, as it remains a significant challenge.

Methylated RNA immunoprecipitation sequencing (MeRIP-seq) is one of the most commonly used tools for quantifying m^6^A modification at a specific site [15]. Several studies have demonstrated that MeRIP-seq can be used to identify novel m^6^A sites and uncover the regulatory mechanism of m^6^A modification on transcription factors [16], [17]. However, there are several drawbacks to this approach. For example, the use of antibodies or enzymes for RNA fragments enrichment may lead to false positive by attaching to non-target RNAs. Furthermore, after RNA fragments are recognized, distinguishing the precise location of m^6^A at single-nucleotide resolution remains challenging [18], [19]. The Oxford Nanopore technology has recently emerged as the potential solution to overcome these limitations through direct sequencing of RNA (dRNA-seq) [20]. dRNA-seq does not require the use of antibodies, and can sequence m^6^A modifications within a very short period of time. Meanwhile, m^6^A sites can be recognized more precisely and with greater resolution at a single-base level [21]. Furthermore, dRNA-seq even enables the detection and characterization of full-length circular RNAs (circRNAs) with greater accuracy and efficiency than previous methods[22], [23].

In our current study, Nanopore dRNA-seq was used to demonstrate for the first time that AZA treatment dramatically reduced m^6^A levels in both bone marrow (BM) of AML patients, and in AML cell line HL-60, indicating AZA exerts its therapeutic effects in AML through its significant demethylation action at the RNA level. Moreover, we discovered differentially m^6^A-modified genes and differentially expressed genes (DEGs) in AML and complete remission (CR) BMs. We also identified three genes with down-regulated m^6^A levels and expression levels as the prognosis-associated genes. These findings were verified by the Gene Expression Omnibus (GEO) clinical data analysis.

## 2 Materials and methods

### 2.1 Patients and samples

BM samples were collected by BM aspiration before and after AZA/VEN treatment from three AML patients who had achieved CR after the treatment in our hospital and then stored at 4 ℃. This study was approved by the Ethics Committee of Beijing Hospital, and all the participants signed informed consent forms.

### 2.2 Nanopore sequencing

The tissues were subjected to total RNA extraction using the TRIzol™ Reagent (Thermo Fisher Scientific, USA) following the manufacturer’s instructions. Subsequently, mRNA was purified from the total RNA using the Dynabeads mRNA Purification Kit (Invitrogen, USA). RNA sequencing was performed using direct-RNA chemistry sequencing kits (SQK-RNA002) (Oxford Nanopore Technologies (Oxford Nanopore Technologies, UK). Nanodrop 2000 and Qubit RNA/dsDNA HS Assay Kits (both from Thermo Fisher Scientific, USA) were used to quantify RNA and hybrid DNA. The Agilent 5200 and DNF-471 RNA kits (both from Agilent, USA) were used to assess the integrity of RNA fragments. Upon completion of the quality checks, libraries were sequenced on a Nanopore sequencing platform GridION. R9.4.1 chips (ONT) were used.

### 2.3 Analysis of m^6^A sites from nanopore reads

All raw signals were basecalled by using Guppy (version 3.3.0) (https://community.nanoporetech.com/downloads) with “--flowcell FLO-MIN106 --kit SQK-RNA002″ option to generate the base sequences. Subsequently, we generated an assembly by aligning the raw signals to the sequences using the re-squiggle algorithm available in Tombo (version 1.5.1) (https://github.com/nanoporetech/tombo). In detail, the “tombo preprocess annotate_raw_with_fastqs” command was applied to add basecalls from a set of FASTQs to raw read files since raw read FAST5 files are required to have basecalls. To achieve a precise alignment between the signal fragment of each base and the reference sequence, the “tombo resquiggle” command with the “-rna” option was utilized. The assembled sequences were aligned to the human genome (GRCh38) using Minimap2 alignment tool (version 2.22) (https://github.com/lh3/minimap2) with the “-ax map-ont” option to align Oxford Nanopore sequencing data with the reference genome (GRCh38). Finally, DENA [24] was used to accurately measure m^6^A levels at single-nucleotide resolution. To be specific, the command “LSTM_extract.py” was used to extract features of new sequences based on known RNA sequences as the first step. Next, the command “LSTM_predict.py” was employed to utilize the pre-trained LSTM neural network model and predict numerical values, which were considered as the number of modified reads for the provided mRNA sequence data. And the m^6^A radio was obtained by dividing the number of modified reads by the number of total reads. We visualized the distribution of m^6^A sites in the human genome using the R package CMplot [25]. Additionally, the sequence logo was created for the m^6^A motifs using the R package ggseqlogo [26].

After the ratio of each m^6^A site was obtained, the sites with an m^6^A ratio of more than 0.5 were considered to have a high m^6^A level. In addition, we computed the difference of m^6^A radio before and after-treatment for each sample pair. A positive value indicates an up-regulation of m^6^A at the site, whereas a negative value indicates a down-regulation. Then, we merged the data of three sample pairs and conducted a paired *t*-test to analyze the significance of their differences. The sites were identified as differentially m^6^A sites if the *P* values were less than 0.1. The resulting differentially m^6^A sites were then intersected with the up- and down-regulated sites in each pair, yielding the common up- and down-regulated m^6^A sites across these three pairs. Finally, to annotate genes corresponding to the detected m^6^A sites, the intersect algorithm in BEDTools (version 2.26.0) was used to obtain differentially m^6^A-modified genes [27].

### 2.4 Analysis of DEGs from nanopore reads

After aligning the Nanopore reads to the human genome (GRCh38) using Minimap2 (version 2.22) with “-N 10″ option to retain at least 10 secondary mappings, we used NanoCount (version 1.0.0) [28] to generate a file containing a normalized gene expression matrix. The counts were employed to compare the gene expression levels between AML and CR BMs. Genes with a fold change (FC) higher than 2 and a *P*-values less than 0.05 were considered as significant DEGs.

### 2.5 Functional enrichment analysis

Functional enrichment analysis was performed for the genes with down-regulated m^6^A modification. Gene Ontology (GO) functional analysis comprising biological process (BP), molecular function (MF), and cellular component (CC) analysis using DAVID [29] (https://david.ncifcrf.gov) and Gene Ontology tools [30] (https://go.princeton.edu). Kyoto Encyclopedia of Genes and Genomes (KEGG) pathway analysis was implemented using DAVID and Enrichr [31], [32] (https://maayanlab.cloud/Enrichr). All significant (GO and KEGG) terms were identified by two tools.

### 2.6 Survival analysis

Kaplan-Meier plots were drawn to show the association between the expression of prognosis-related genes and the overall survival (OS) of AML patients, and the result were analyzed by using the log-rank test. The online tool Kaplan-Meier Plotter [33] (http://kmplot.com) was used to identify the prognostic values of these three genes.

### 2.7 Relative quantification of residue specific m^6^A methylation

For the m^6^A-retrotranscription reaction, 75 - 100 ng of RNA, 100 nM of each primer, 50 μM of dNTPs and 0.1 U of BstI (NEB) or 0.8 U of MRT (ThermoScientific) were used. The cycling conditions were as follows: 50 °C for 15 min, 85 °C for 3 min, and maintenance at 4 °C. For the qPCR, 1.5 μl of the retrotranscription reaction was used together with 100 nM of each primer and 2X SYBR green (BioRad). The cycling conditions were as follows: 95 °C for 30 s, and 50 cycles x (95 °C for 15 s, 58 °C for 30 s) followed by the melting curve analysis using the follow condition: 95 °C for 10 s and 65 °C (1 min) - 95 °C (15 s) with a 0.5 °C increment [34]. The sequences of all the primers are available in Table S1.

### 2.8 Statistical analysis

The SPSS 26.0 software (SPSS Inc., Chicago, IL, USA) was used for statistical analysis. Two groups were compared using independent samples *t*-test or Mann-Whitney U test of variance. The paired samples were subjected to a Paired Samples *t*-test for analysis. A *P* value of < 0.05 was considered statistically significant.

## 3 Results

### 3.1 Patient characteristics

Three frail elderly AML patients were enrolled, whose IACA-AML index scored 3 according to our previous studies [35]. All these three patients were determined as at high-risk and received VEN/AZA as induction chemotherapy. The characteristics of these patients are presented in Table 1. All of them achieved CR, among whom two patients achieved persistent leukemia-free survival at the latest follow-up visits and one patient relapsed after 6 cycles of consolidation chemotherapies and died 24 days after relapse.

**Table 1:** The characteristics of three patients in our study. VEN: venetoclax, AZA: azacytidine.

No	Gender	Age	Comorbidities	WBC at diagnosis (×10^9^/L)	Blast in BM at diagnosis	Genetic mutation at diagnosis	Cytogenetics at diagnosis	IACA-AML index	Induction chemotherapy	Complete remission (CR) after induction chemotherapy	Blast in BM at CR	Relapse during follow-up	Survival at last follow-up
1	Female	74	Hypertension	1.55	61%	WT1	Normal	3	VEN+AZA	Yes	2%	No	Leukemia-free survival, 438 days after CR
2	Male	79	None	0.77	63%	None	Normal	3	VEN+AZA	Yes	2%	No	Leukemia-free survival, 445 days after CR
3	Male	63	Coronary heart disease; atrial fibrillation; tricuspid incompetence; Left nephrectomy; perianal abscess	49.94	63%	RUNX1	Normal	3	VEN+AZA	Yes	4%	Yes, 235 days after CR	Died from relapse, 24 days after relapse

### 3.2 Characteristics of m^6^A sites in AML and CR BMs

**Fig. 1:**
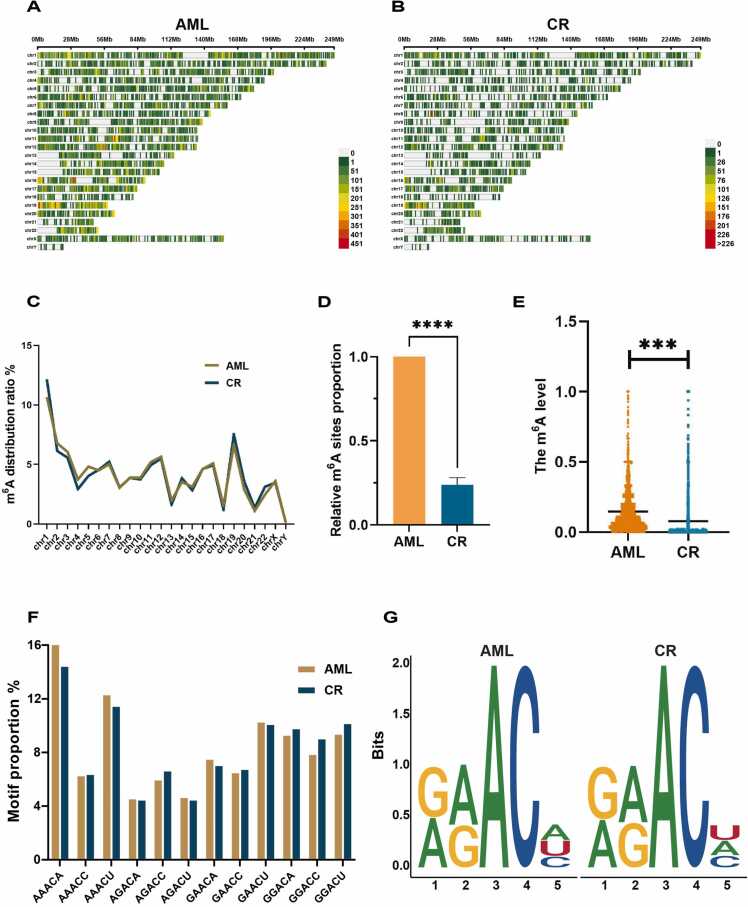
The distribution characteristics of m^6^A sites in AML and CR BMs. **A-B**. The distribution of m^6^A sites in AML and CR BMs throughout the genome. The color scale from green to red represents the density of m^6^A level. **C**. The distribution ratio of m^6^A sites on each chromosome in AML (yellow) and CR (blue) BMs. **D**. Relative m^6^A sites proportion of AML (orange) and CR (blue) BMs quantified by the amount of AML BMs. * ** * *P* < 0.0001. **E**. The m^6^A levels of AML (orange) and CR (blue) BMs. * ** *P* < 0.001 **F**. The proportion of each m^6^A motif in the AML (yellow) and CR (blue) BMs. **G**. The sequence logos revealed a single base variation in the enrichment of m^6^A motifs between AML (left) and CR (right) BMs.

We analyzed the distribution of m^6^A sites in both AML and CR BMs. As shown in Fig. 1A and B, the m^6^A sites were widely distributed throughout the genome, and the distribution ratio of m^6^A sites on each chromosome was approximately the same in both groups (Fig. 1C), suggesting that AZA/VEN treatment had a wide range of effects throughout the whole genome, and the distribution of m^6^A sites and genes was not entirely consistent (Fig. S1). However, it was visually observed that the number of m^6^A sites significantly decreased after AZA/VEN treatment (Fig. 1B). Quantification of the m^6^A sites revealed that the relative amount of m^6^A sites in CR BMs was about a quarter of that in AML BMs (Fig. 1D). Correspondingly, the m^6^A levels in AML BMs was also significantly higher than that in CR BMs (Fig. 1E). The same results were also found in AML cell line HL-60 treated with AZA at IC_50_ concentrations (Fig. S2A-B), suggesting AZA had a significant demethylation effect at the RNA level. In addition, the m^6^A sites in both AML and CR BMs exhibited a nearly uniform distribution across all RRACH motifs. In both tissues, the m^6^A sites were mostly aggregated on the “AAACA” motif, with “AGACA” being the least abundant (Fig. 1F). Sequence logo analysis showed that only one base differed between AML and CR BMs in the RRACH motifs (Fig. 1G). Therefore, compared with AML BMs, the CR BMs had a significantly decreased m^6^A level, but the distribution pattern of m^6^A on chromosomes and motifs was not greatly affected.

### 3.3 Down-regulated m^6^A sites and genes after AZA/VEN treatment

**Fig. 2:**
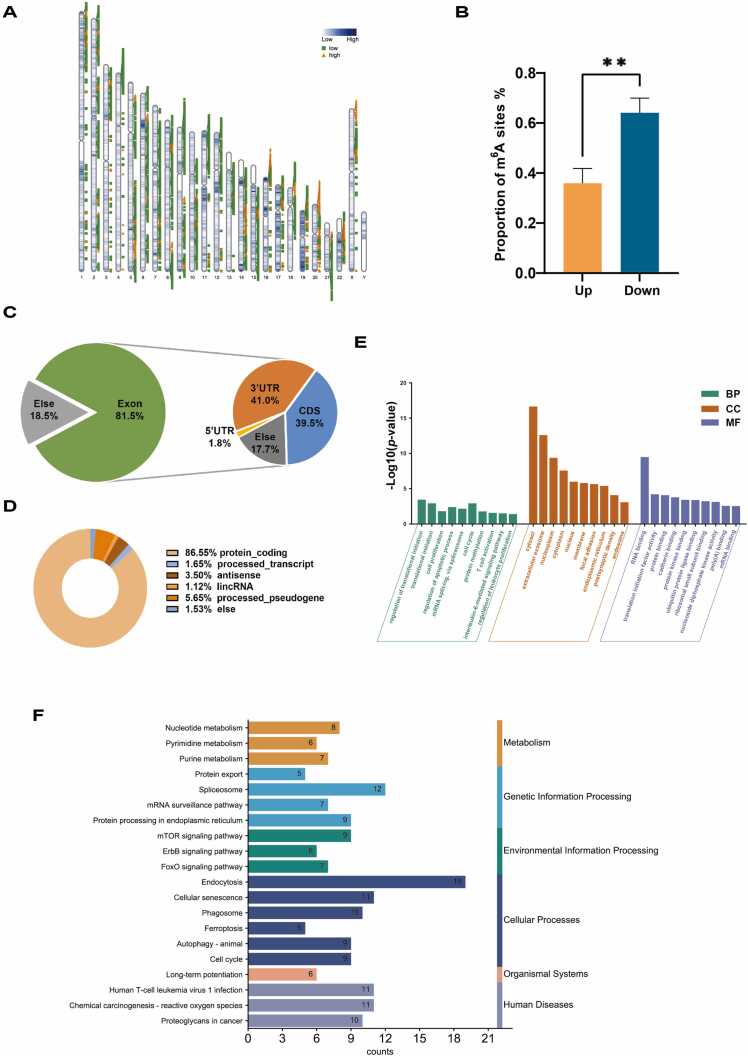
The analysis of differentially m^6^A-motified genes. A. Density heatmaps of the distribution of differentially m^6^A sites along chromosomes between AML and CR BMs. The green squares represent down-regulated m^6^A sites and the orange triangles represent up-regulated m^6^A sites. The darker colors show a greater density of the m^6^A sites. B. Up- and down-regulated proportions of m^6^A sites and genes. C. The distribution of down-regulated m^6^A sites within functional regions of genes. D. The types of genes exhibiting down-regulated m^6^A modifications. E. The top 10 terms in GO enrichment for down-regulated m^6^A-motified genes. F. The top 20 pathways in KEGG enrichment for down-regulated m^6^A-motified genes.

The up and down-regulated differentially m^6^A sites were widely distributed across the chromosomes in AML and CR BMs, with the exception of Y chromosome (Fig. 2A). The majority of the differentially m^6^A sites were down-regulated (Fig. 2B). Consistently, the genes with reduced m^6^A accounted for the larger proportion. In addition, KEGG functional enrichment analysis of m^6^A up-regulated genes revealed no pathway associated with AML (Fig. S3A). Therefore, our subsequent investigations focused on the sites and genes in CR BMs with down-regulated m^6^A level compared with AML BMs. To identify the m^6^A down-regulated sites, we performed the paired *t*-test on three sample pairs and screened out m^6^A sites with a *P* value of < 0.1. By intersecting these significantly different m^6^A sites with the sites where m^6^A level decreased in each pair of samples, we obtained significantly different sites with down-regulated m^6^A level in all three pairs.

Next, the distribution of the m^6^A down-regulated sites in different functional regions of the genes was analyzed. As shown in Fig. 2C, the majority of these sites were found in the exonic regions of the genome, especially in the 3′ untranslated regions (3′ UTRs), where they played a dominant role in post-transcriptional regulation and translation, as previous observed [36]. Furthermore, 86.55% of genes with reduced m^6^A levels in the CR BMs were protein-coding genes (Fig. 2D), suggesting that they were mainly involved in RNA translation.

To understand the functional roles of m^6^A in AML, we performed GO enrichment analysis for the genes with down-regulated m^6^A modifications. The analysis evaluated three subontologies: biological process (BP), molecular function (MF), and cellular component (CC) (Fig. 2E). BP showed that genes were enriched in the regulation of translation, apoptosis, mRNA splicing, protein methylation, regulation of leukocyte proliferation, and some AML-related terms, demonstrating the importance of these genes with down-regulated m^6^A modifications in controlling RNA translation and tumor development (Fig. 2E). With regard to MF, the genes with down-regulated m^6^A modifications were also clustered in mRNA binding, poly(A) binding, protein binding, and translation initiation factor activity (Fig. 2E). The KEGG pathway enrichment analysis revealed nucleotide metabolism, mRNA splicing, protein processing and AML-associated signaling pathways (Fig. 2F). Although no pathway was enriched in AML, some AML-related genes such as *PTEN*, *FOS*, *CALR*, and *CD44* were found in the human disease pathways [37], [38], [39], [40].

### 3.4 Identification of genes with prognostic value

**Fig. 3:**
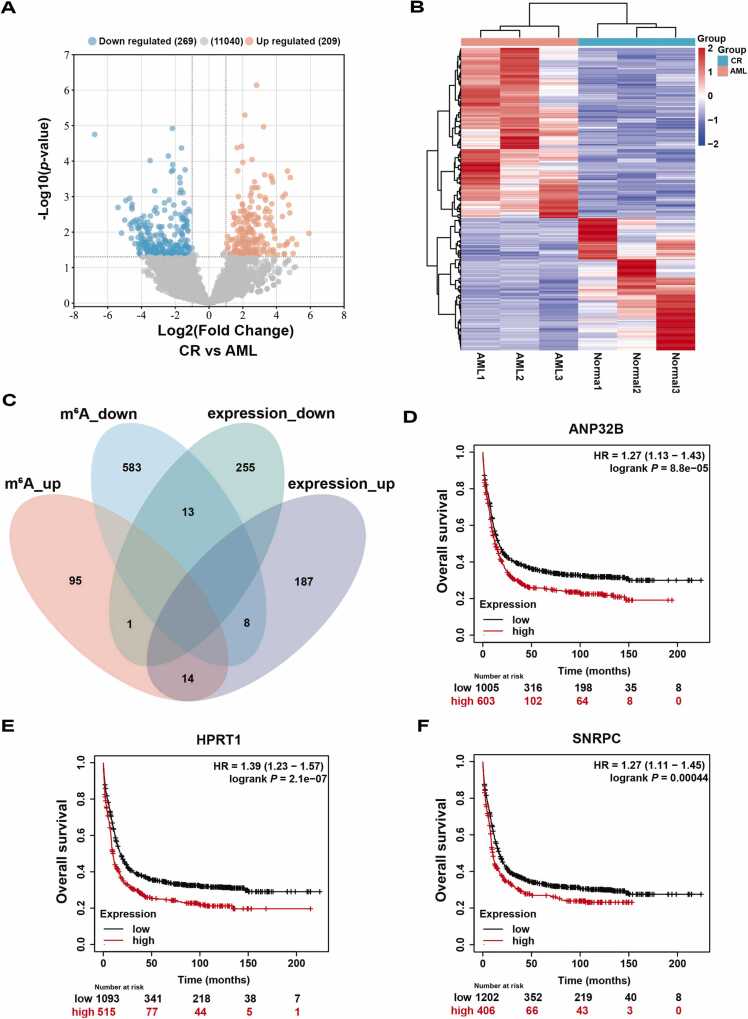
The analysis of down-regulated m^6^A-modified and expressed genes. A. Volcano plot for the comparison of the DEGs between the AML and CR BMs. The cutoff values Log2(fold change) > 1 and -log10(*P*-value) < 0.05 were used to identify DEGs. Non-changed genes are shown in grey color. Red color is indicative of up-regulated genes and blue is indicative of down-regulated genes. B. The heatmap of the DEGs between AML and CR BMs. C. The Venn diagram shows the intersection number of differentially m^6^A-modified and expressed genes. D-F. Overall survival curves of three prognosis-related genes. The curves were derived from a total of 1608 AML patients from 5 GSE databases (GSE1159, GSE12417, GSE37642, GSE6891 and GSE8970) by Kaplan-Meier Plotter.

To determine the potential value of the genes in predicting the OS of AML patients, dRNA-seq was performed to analyze the data from the Nanopore sequencing libraries. A total of 478 DEGs, including 209 up-regulated and 269 down-regulated genes, were found to be statistically significant in CR BMs compared with AML BMs (|log fold change (logFC)| > 1, *P* < 0.05) (Fig. 3A). The heatmap generated from the analysis of DEGs showed that the expression patterns of these identified DEGs could accurately discriminate between these two sample types (Fig. 3B). And then, 110 m^6^A up-regulated and 604 m^6^A down-regulated genes were identified, of which 2/3 of the m^6^A down-regulated genes were prognostic genes (Fig. S4). We conducted a comprehensive analysis by integrating the transcriptome and m^6^A methylome data, during which we compared the genes that exhibited differentially m^6^A-modified with the DEGs, thereby identifying genes that overlapped between these two datasets (Fig. 3C). As the upregulated genes were mostly associated with virus infection pathways (Fig S3B), we focused on 13 genes with decreased m^6^A and expression levels (Fig. S5), among which three genes (i.e., *HPRT1*, *SNRPC*, and *ANP32B*) that were found to be significantly associated with the prognosis of AML patients were ultimately selected (Fig. 3 D-F). The OS curves revealed that the decreased expression levels of these three genes were linked to a favorable prognosis.

### 3.5 Analysis of three prognosis-related genes

**Fig. 4:**
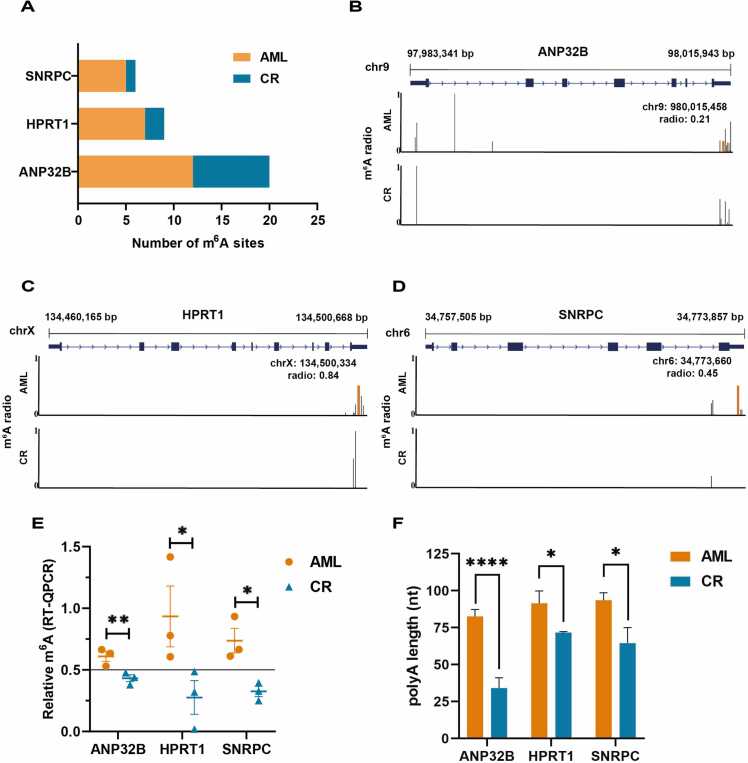
The analysis of three prognosis-related genes. A. Comparison of the total m^6^A sites number of three prognosis-related genes (*HPRT1*, *ANP32B*, and *SNRPC*) in the AML (orange) and CR (blue) BMs. B-D. The distribution of identified m^6^A sites within the three genes associated with prognosis. Each orange line represents an m^6^A site that differs significantly across all three sample pairs, and black lines represent m^6^A sites that differ in one/two sample pairs. E. Relative amount of the m^6^A levels in three prognosis-related genes, where a *P* values above 0.5 are considered positive for methylation and below 0.5 are considered as negative for methylation. F. Comparison of polyA tails of mRNA in AML (orange) and CR (blue) BMs. * *P* < 0.05, * * *P* < 0.01, * ** * *P* < 0.0001.

All of the m^6^A sites of these three genes were markedly decreased in CR BMs when compared to AML BMs (Fig. 4A), primarily within the 3′ UTR region. Furthermore, we identified one site within each gene that displayed significant differences across all three pairs, with all of them being concentrated in the 3′ UTR region (Fig. 4 B-D). In addition, as revealed by qPCR, the number of m^6^A sites in these three prognosis-related genes was smaller in CR BMs [34] (Fig. 4E). As m^6^A was associated with mRNA stability and could facilitate mRNA translation [41], [42], [43], we investigated the polyA tail length to validate the mRNA stability; as anticipated, the polyA tail of all three prognosis-related genes was significantly shorter in CR BMs than in AML BMs (Fig. 4F).

## 4 Discussion

In recent years, there has been growing interest in m^6^A, the most common internal modification of RNA in eukaryotic cells. The m^6^A plays a crucial role in various aspects of RNA metabolism, including mRNA splicing, nuclear export, translation, and mRNA stability [44]. Evidence indicates that dysregulation of m^6^A can impact tumorigenesis by modulating the expression of various targets at the post-transcriptional level, primarily by affecting mRNA stability and translation [45]. In addition, AML involves DNA methylation because abnormal gene activities, such as the silencing of tumor suppressor genes and growth-regulatory genes, occur during leukemic transformation through epigenetic inactivation via DNA hypermethylation. [46]. AZA is a DNA demethylation agent that can suppresses the catalytic activity of DNA methyltransferase, reduce the level of DNA methylation modification in cancer cells, and thus restore the expression of tumor suppressor genes and exert antitumor activities. It has been approved to treat AML, particularly in elderly patients who are not eligible for chemotherapy [47], [48]. AZA also exhibits similar effects on RNA because AZA is a ribonucleoside and its primary target may be cellular RNA rather than DNA [49]. However, the majority of the currently available studies of AZA have focused on its effect on DNA methylation levels, and there is a lack of the global landscape of m^6^A. Therefore, in our currently study, we used Nanopore direct RNA-seq to assess changes in the m^6^A level in elderly AML patients following AZA/VEN treatment.

First of all, there was a significant reduction in the abundance of m^6^A sites and m^6^A levels throughout the entire genome of AML patients following AZA/VEN treatment. AZA plays the predominate demethylating role, whereas VEN is primarily a B-cell lymphoma 2 (BCL-2) inhibitor that selectively inhibits the survival signaling pathway of leukemia cells[7], [50]. To examine the impact of demethylation drug on the RNA methylation patterns in leukemic cells, AZA was selected to treat HL-60 alone at IC_50_ concentrations. The results showed a significant decrease in m^6^A levels in HL60 cells (Fig S2B). This conclusion was also supported by the visual observation of the color intensity of each line segment representing the level of m^6^A modification (Fig S6A-B). However, there was no significant difference in the number of m^6^A sites in the cells (Fig S6A-B). Due to the IC_50_ concentration of AZA used, which is insufficient to achieve complete reversal of hypermethylation in HL-60, we did not observe a significant decrease in the number of m^6^A sites in cells after AZA treatment. However, the overall reduction in m^6^A levels also indicated the significant demethylation effect of AZA at the RNA level in the treatment of AML. Despite the significant variances in the number of m^6^A sites and m^6^A levels between these two groups, their chromosome and motif distributions exhibited considerable similarities. However, due to the small sample size and limited m^6^A sites on the Y chromosome, no distinct differential m^6^A sites were identified on the Y chromosome. Furthermore, most current research efforts focused on the m^6^A regulators, including "writers", "erasers", and "readers" along with their expression and functions in AML [51], [52], [53]. These regulators play essential roles in the addition, removal, and recognition of m^6^A modifications. As the landscape of genome-wide m^6^A methylome of AML patients and their significance in the development of AML are poorly illustrated, our current study bridged this gap.

As a demethylating drug, AZA reduces m^6^A levels on known leukemogenic mRNAs, leading to a decrease in their expression. This may suppress AML growth and induce differentiation and apoptosis [54]. In our present study, three prognosis-related genes (i.e., *HPRT1*, *ANP32B,* and *SNRPC*) were identified to be associated with favorable outcomes among genes exhibiting a significant reduction in both m^6^A modification and expression. Studies have validated that *HPRT1* and *ANP32B* were protective factors for AML patients. *HPRT1* is a major metabolic enzyme for GMP salvage synthesis and can be screened by survival analysis with the data from TCGA and GEO databases [55]. *ANP32B*, a member of ANP32 family proteins, is mainly involved in the regulation of gene transcription, and the knockdown of *ANP32B* has been observed to facilitate apoptosis and hinder cell proliferation in AML cells [56]. Despite the lack of studies specifically focusing on the relationship between *SNRPC* and AML, evidence suggests that the product of this ribosome-related gene was involved in mRNA splicing and protein synthesis [57] and served as a tumor suppressor gene in various tumors such as hepatocellular carcinoma [58] and ovarian cancer [59]. In our present study, we identified these three genes that can serve as a novel set of biomarkers for evaluating the therapeutic efficacy of AZA/VEN in treating AML.

Surprisingly, transcriptome analysis identified *YTHDF1* as the sole m^6^A reader exhibiting differential expression in CR BMs, with a significant reduction of expression observed (Table S2). Previous studies have illustrated YTHDF1 could recruit translation initiation factors to promote mRNA translation by binding the m^6^A sites in 3′ UTR, and the role of YTHDF1 in mRNA stability could not be completely excluded in this process [60], [61]. Thus, we assume that the reduction of m^6^A levels leads to a decrease in mRNA stability due to the lack of YTHDF1, resulting in the down-regulation of these three prognosis-related genes (*HPRT1* and *SNRPC* showed significant correlations with *YTHDF1*, as seen in Fig. S7). Due to the relatively low protein content in the BM used in our project, we validated the mRNA stability by examining the length of poly(A) tails. The length of poly(A) tails is closely associated with mRNA stability, and longer poly(A) tails are believed to correspond to greater mRNA stability [62]. It was found in our study that the poly(A) tails of these three genes were significantly shorter in CR BMs than in AML BMs.

In conclusion, our research has provided the initial insight into the global landscape of m^6^A levels in both AML and CR BMs and indicated that m^6^A levels in the BMs during the AML phase is at a relatively high condition. AZA also has a demethylation effect at the RNA level in AML patients. Three prognosis-related genes (*HPRT1*, *ANP32B*, and *SNRPC*) may serve as a set of potential biomarkers for evaluating the therapeutic efficacy of AZA/VEN treatment in AML patients.

## Ethics statement

The studies involving human participants were reviewed and approved by the Ethics Committee of Beijing Hospital (Approval Number: [2022BJYYEC-189–02]).

## Funding

This work was supported by National High Level Hospital Clinical Research Funding (BJ-2022-169), CAMS Innovation Fund for Medical Sciences (2021-I2M-1-050).

## CRediT authorship contribution statement

**Zaifeng Zhang**: Formal analysis, Investigation, Software, Visualization, Writing – original draft. **Lili Zhang**: Software. **Jiangtao Li**: Resources. **Ru Feng**: Resources. **Chang Li**: Software. **Ye Liu**: Formal analysis. **Gaoyuan Sun**: Investigation. **Fei Xiao**: Conceptualization, Funding acquisition, Methodology, Project administration, Supervision, Writing – review & editing. **Chunli Zhang**: Conceptualization, Funding acquisition, Investigation, Project administration, Resources, Supervision, Writing – review & editing.

## Declaration of Competing Interest

The authors have declared no competing financial interests.

## Appendix A Supplementary material

Table S1. Primer sequence information.

.

Table S2. DEGs in CR BMs compared with AML BMs.

.

**Fig. S1.** The distribution of m^6^A sites and genes in tumor tissue throughout the genome. The red bars represent the gene density, and the blue bars represent the m^6^A methylation site density. The distribution of these two sets is not completely consistent.

.

**Fig. S2.** The m^6^A levels of HL-60 treated with AZA at IC_50_ concentration. **A**. The IC_50_ concentration of AZA for HL-60, IC_50_ = 1.86 μM. **B**. The m^6^A levels of DMSO (orange) and AZA (blue) treated HL-60, * ** *P* < 0.001.

.

**Fig. S3.** The KEGG analysis for genes with up-regulated m^6^A modification and up-regulated expression respectively A: The KEGG enrichment analysis for up-regulated m^6^A-motified genes. B: The KEGG enrichment analysis for up-regulated expression genes.

.

**Fig. S4.** The analysis of the prognostic significance of genes with down-regulated m^6^A modifications.

.

**Fig. S5.** The specific expression levels and methylation levels of the 13 genes with decreased expression and m^6^A levels. **A**. The expression levels of these 13 genes. **B**. The m^6^A levels of the 13 genes.

.

**Fig. S6.** The distribution characteristics of m^6^A sites in DMSO and AZA treated HL-60. **A-B.** The distribution of m^6^A sites in HL-60 with DMSO or AZA treatment throughout the genome. The color scale from green to red represents the density of m^6^A level.

.

**Fig. S7.** The correlation analysis of gene expression. The expression of YTHDF1 highly correlated with the that of SNRPC and HPRT1, respectively.
