# Supplementary material for: Comprehensive analysis of m6A methylome alterations after azacytidine plus venetoclax treatment for acute myeloid leukemia by nanopore sequencing
Source: Comput Struct Biotechnol J. 2024 Mar 2;23:1144–53. doi: 10.1016/j.csbj.2024.02.029 (PMC10950754; doi:10.1016/j.csbj.2024.02.029)
Supplement: Supplementary file 1 — Table S1. Primer sequence information. [file mmc1.docx]

Primer sequences (5’-3’):

*ANP32B*+: TCCATGAGCAGTCCAACCAATACTGAACAG

*ANP32B*-: CTGCAGGTCATCTGGGGTCTTAATCATC

*ANP32B*-F: TGCTTTTCTTGGTTCTGTGGT

*ANP32B*-R: CTTTAGGACAGTGGAAAGGCA

*HPRT1*+: AGTTCAATGTTTCACTCAATAGTGCTGTGG

*HPRE1*-: CAAGTTAAACAACAATCCGCCCAAAGGG

*HPRT1*-F: TCCTATTGACATCGCCAGTAAA

*HPRT1*-R: GGCTCATAGTGCAAATAAACAGT

*SNRPC*+: AGGACAGGGGAGCAAGTCTTCCTTATGCTG

*SNRPC*-: TCAGTATCACAGCAGCATGATCTCCTGGTG

*SNRPC*-F: CCTCTTCTTTGTTTTGTCCTGC

*SNRPC*-R: CCTTATCTGTCTGGTCGAGTCA
