# Supplementary figures and images for: Comprehensive analysis of m6A methylome alterations after azacytidine plus venetoclax treatment for acute myeloid leukemia by nanopore sequencing
Source: Comput Struct Biotechnol J. 2024 Mar 2;23:1144–53. doi: 10.1016/j.csbj.2024.02.029 (PMC10950754; doi:10.1016/j.csbj.2024.02.029)

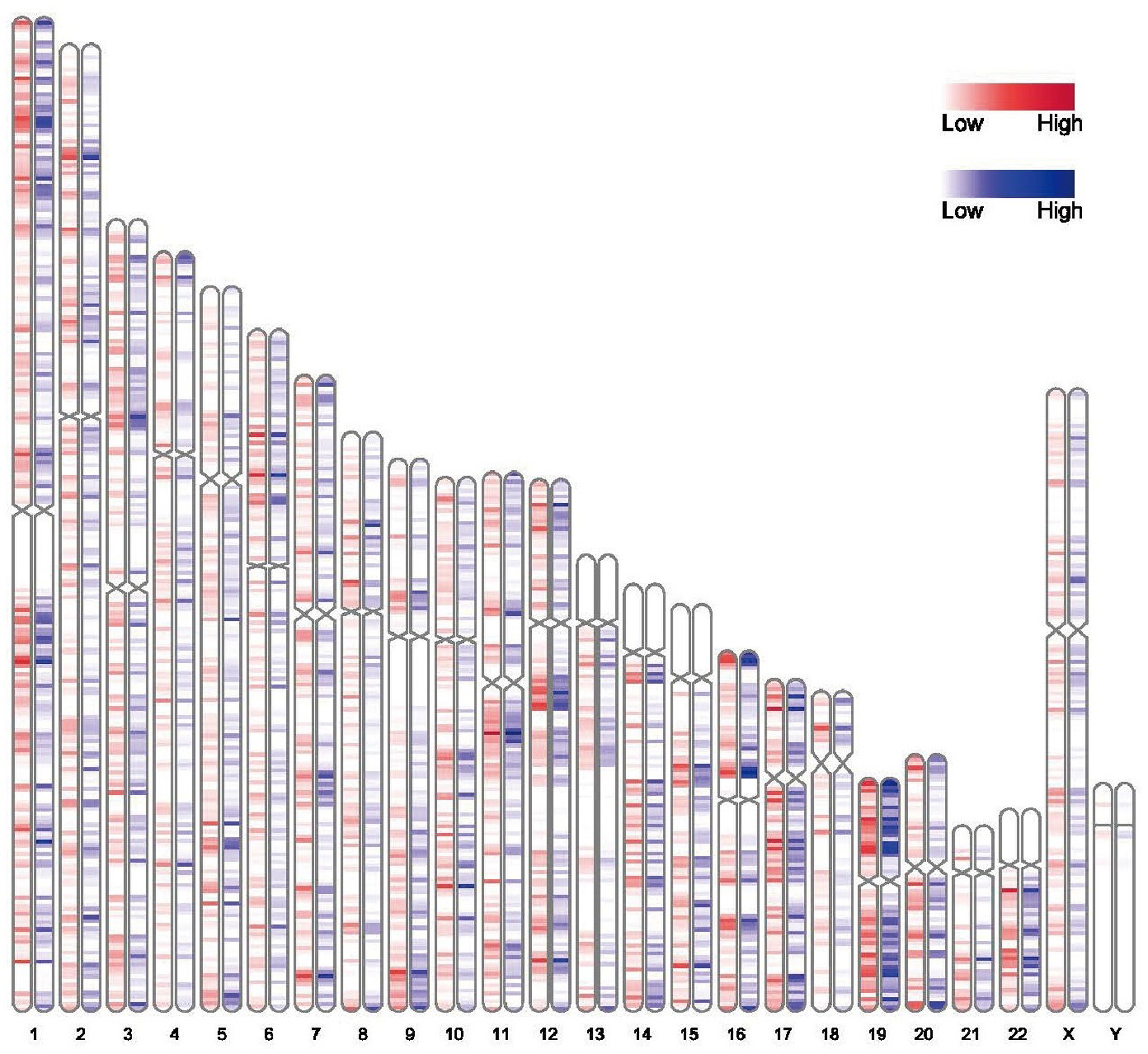

Supplement: Supplementary file 3 — Fig. S1. The distribution of m6A sites and genes in tumor tissue throughout the genome. The red bars represent the gene density, and the blue bars represent the m6A methylation site density. The distribution of these two sets is not completely consistent. [file mmc3.jpg]

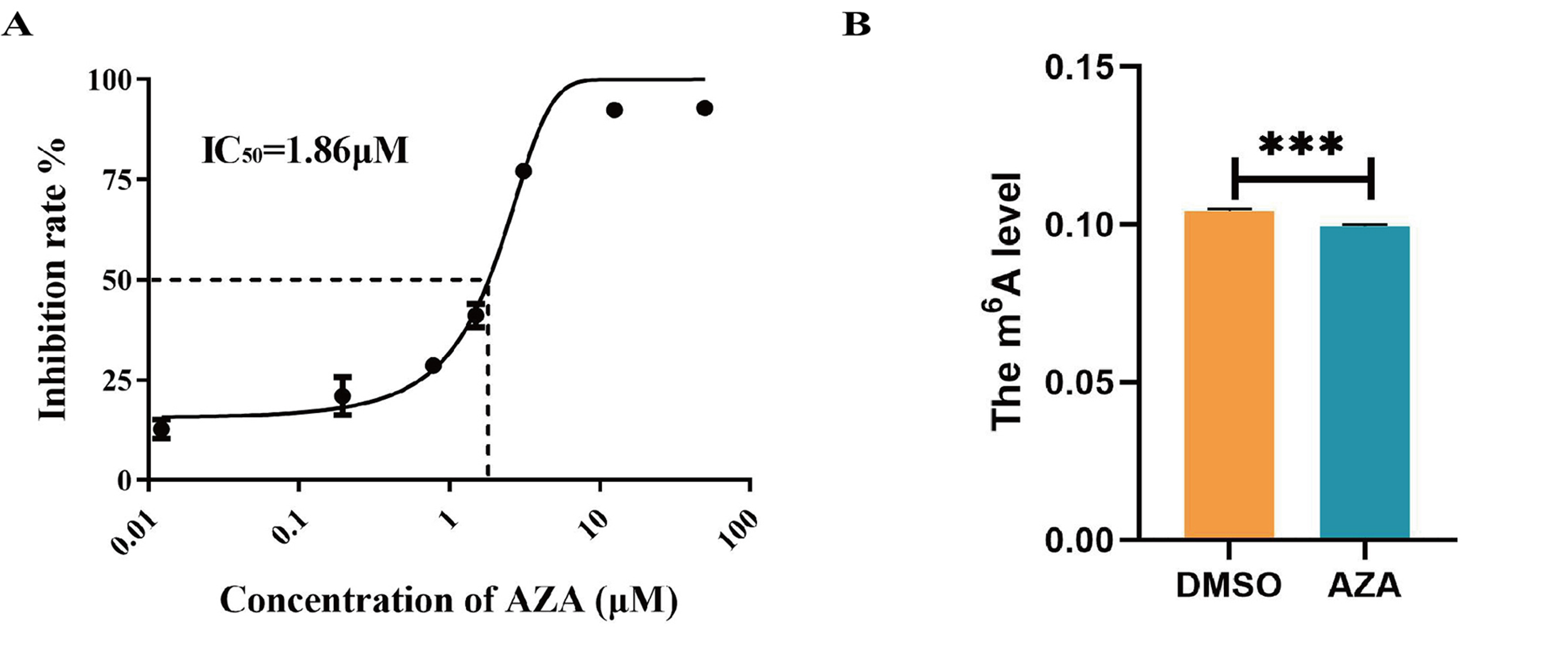

Supplement: Supplementary file 4 — Fig. S2. The m6A levels of HL-60 treated with AZA at IC50 concentration. A. The IC50 concentration of AZA for HL-60, IC50 = 1.86 μM. B. The m6A levels of DMSO (orange) and AZA (blue) treated HL-60, * ** P < 0.001. [file mmc4.jpg]

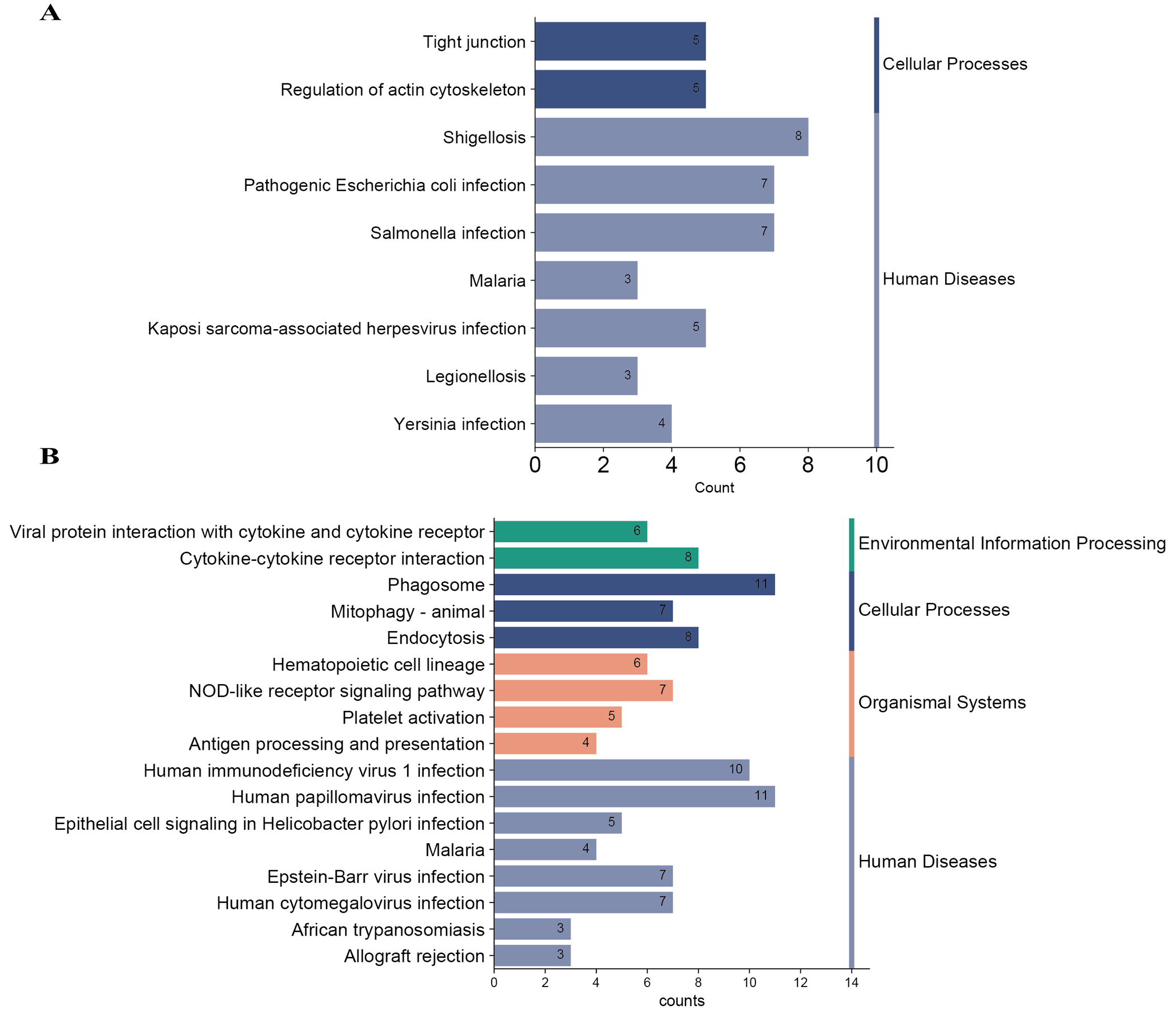

Supplement: Supplementary file 5 — Fig. S3. The KEGG analysis for genes with up-regulated m6A modification and up-regulated expression respectively A: The KEGG enrichment analysis for up-regulated m6A-motified genes. B: The KEGG enrichment analysis for up-regulated expression genes. [file mmc5.jpg]

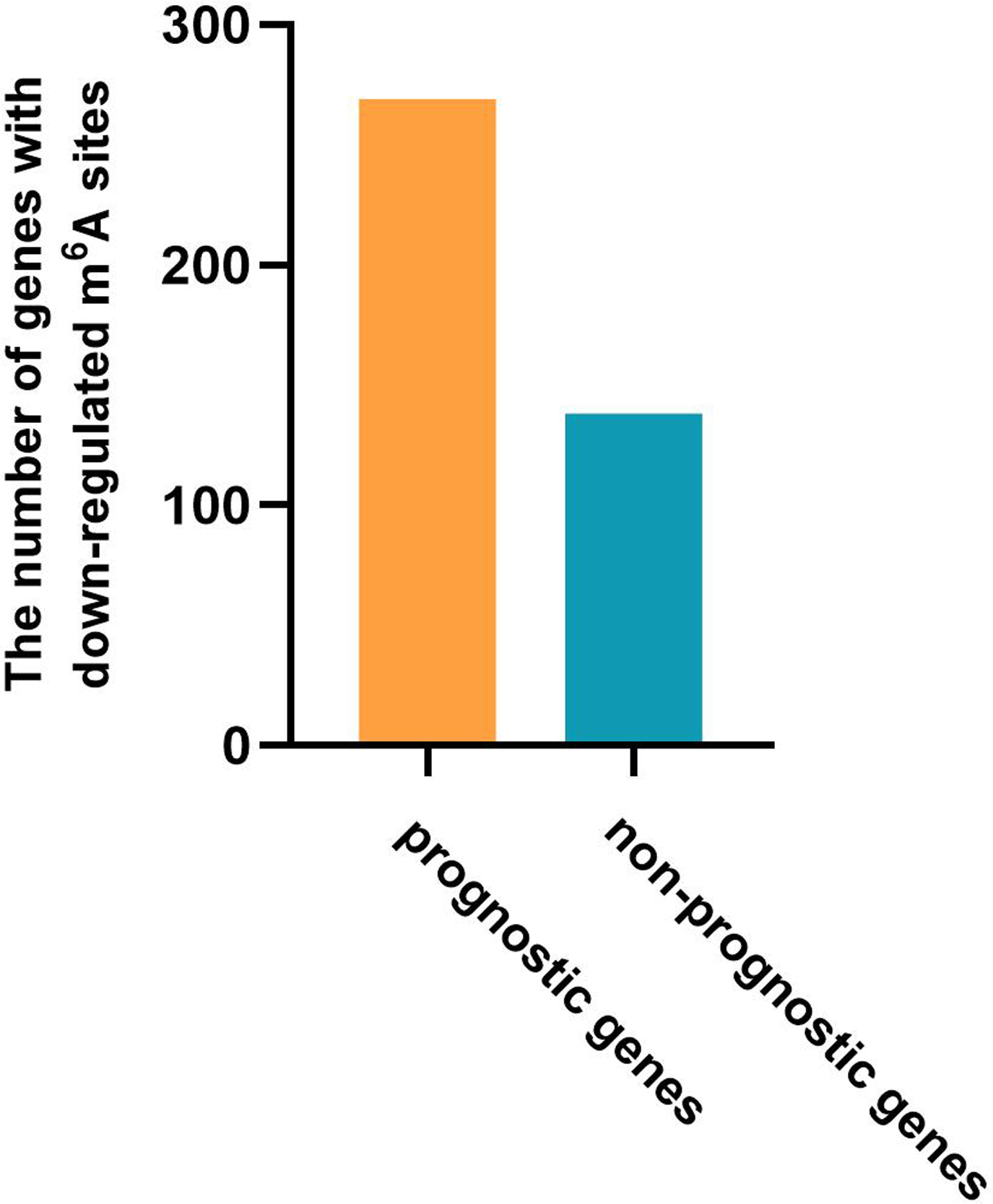

Supplement: Supplementary file 6 — Fig. S4. The analysis of the prognostic significance of genes with down-regulated m6A modifications. [file mmc6.jpg]

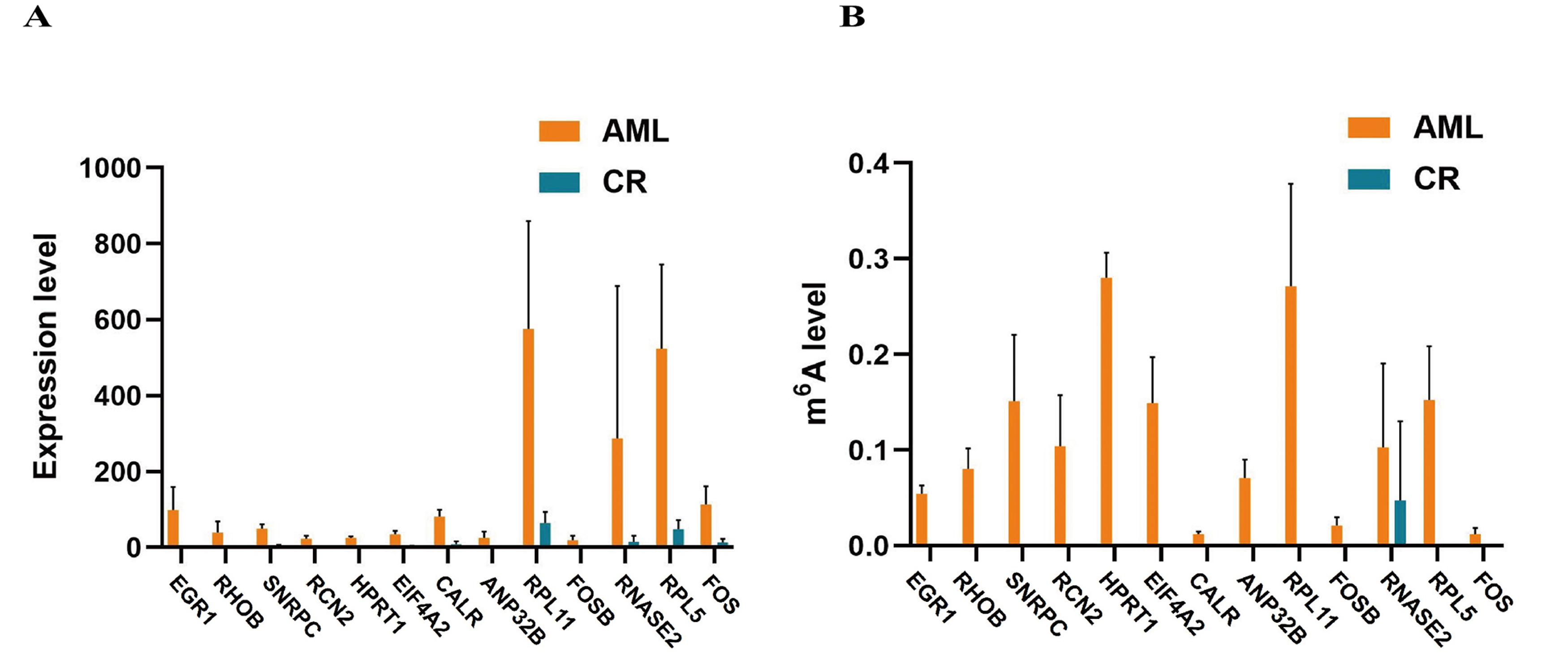

Supplement: Supplementary file 7 — Fig. S5. The specific expression levels and methylation levels of the 13 genes with decreased expression and m6A levels. A. The expression levels of these 13 genes. B. The m6A levels of the 13 genes. [file mmc7.jpg]

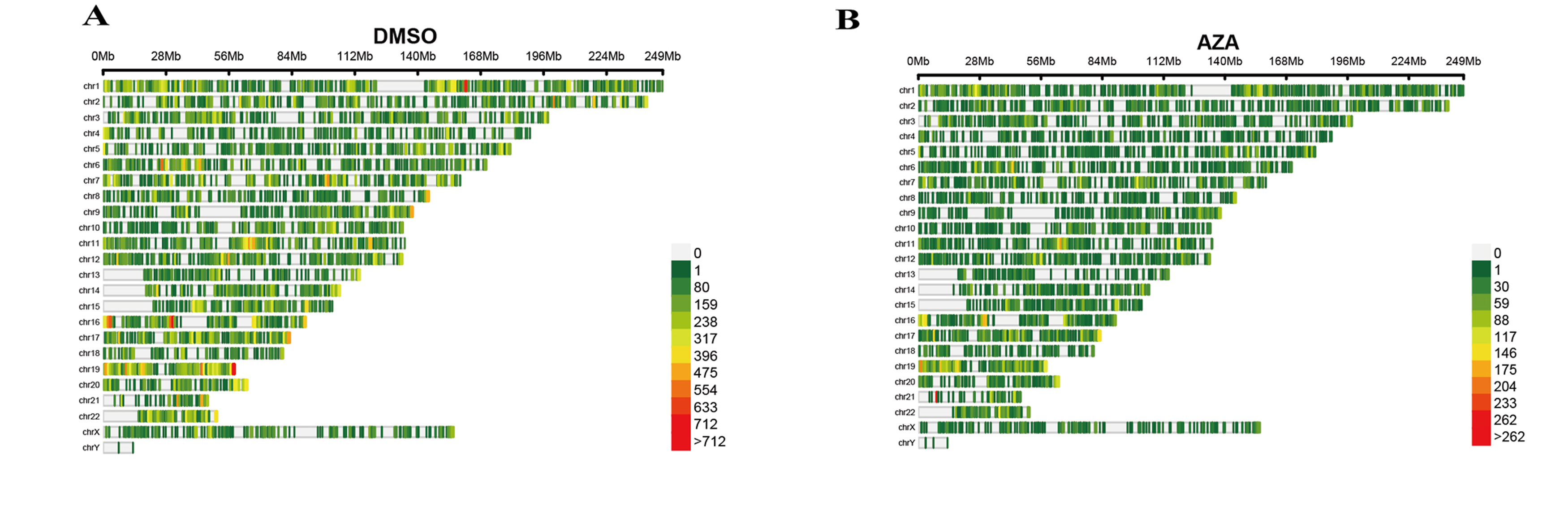

Supplement: Supplementary file 8 — Fig. S6. The distribution characteristics of m6A sites in DMSO and AZA treated HL-60. A-B. The distribution of m6A sites in HL-60 with DMSO or AZA treatment throughout the genome. The color scale from green to red represents the density of m6A level. [file mmc8.jpg]

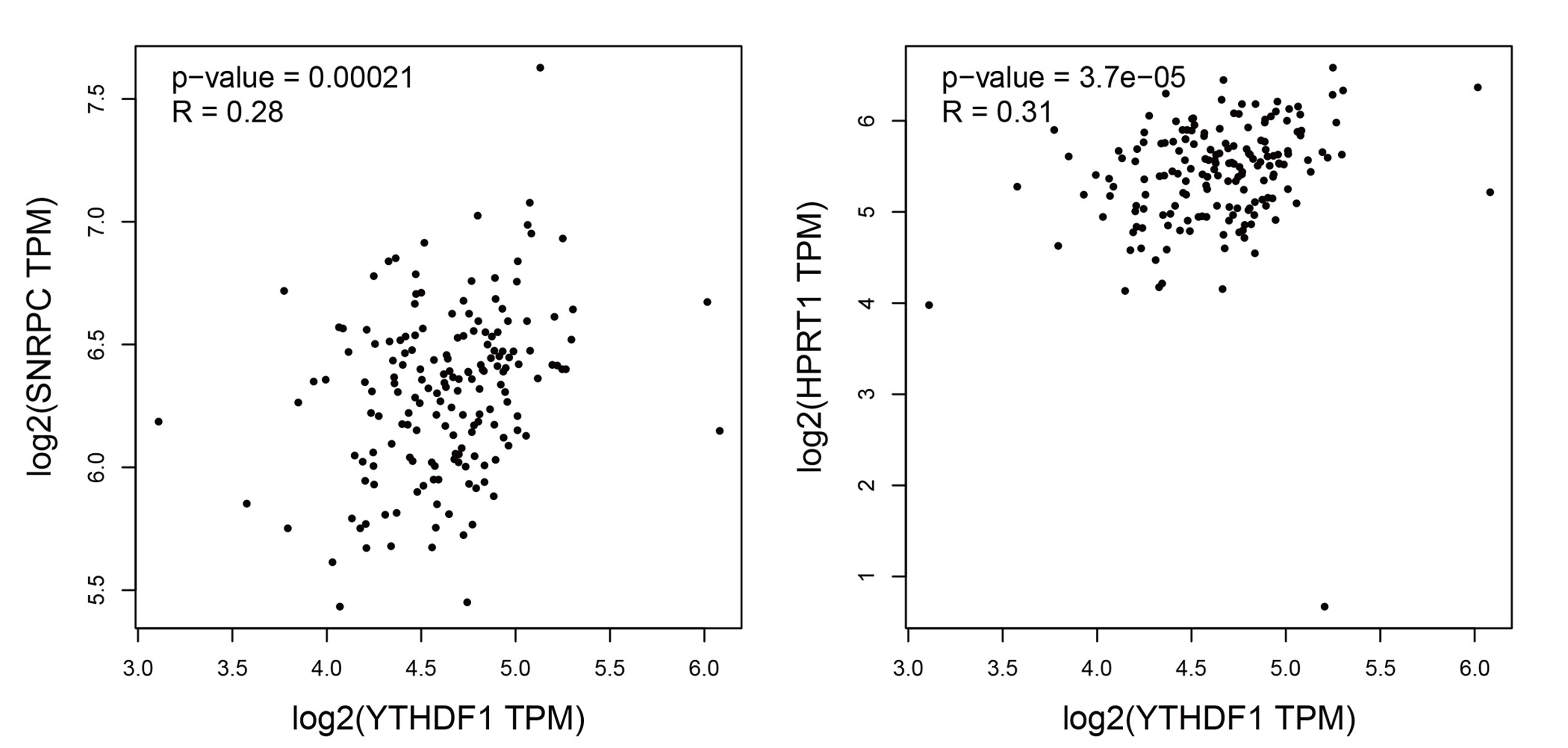

Supplement: Supplementary file 9 — Fig. S7. The correlation analysis of gene expression. The expression of YTHDF1 highly correlated with the that of SNRPC and HPRT1, respectively. [file mmc9.jpg]
